# The Expression Level and Prognostic Value of Y-Box Binding Protein-1 in Rectal Cancer

**DOI:** 10.1371/journal.pone.0119385

**Published:** 2015-03-19

**Authors:** Yu Zhang, Ping-Wu Zhao, Gang Feng, Gang Xie, An-Qun Wang, Yong-Hong Yang, Dong Wang, Xiao-Bo Du

**Affiliations:** 1 Department of Oncology, MianYang Central Hospital, MianYang, People’s Republic of China; 2 Department of Surgery, LuZhou Medical College, LuZhou, People’s Republic of China; 3 Department of Surgery, MianYang Central Hospital, MianYang, People’s Republic of China; 4 Department of Pathology, MianYang Central Hospital, MianYang, People’s Republic of China; Sapporo Medical University, JAPAN

## Abstract

The aims of this study were to simultaneously evaluate the expression of Y-box binding protein-1 (YB-1) in non-neoplastic rectal tissue and rectal cancer tissue, and to collect clinical follow-up data for individual patients. Additionally, we aimed to investigate the developmental functions and prognostic value of YB-1 in rectal cancer. We performed immunohistochemical studies to examine YB-1 expression in tissue samples from 80 patients with rectal cancer, 30 patients with rectal tubular adenoma, and 30 patients with rectitis. The mean YB-1 histological scores for rectal cancer, rectal tubular adenoma, and rectitis tissue specimens were 205.5, 164.3, and 137.7, respectively. Shorter disease-free and overall survival times were found in patients with rectal cancer who had higher YB-1 expression than in those with lower expression (38.2 months vs. 52.4 months, P = 0.013; and 44.4 months vs. 57.3 months, P = 0.008, respectively). Our results indicate that YB-1 expression is higher in rectal cancer tissue than in rectal tubular adenoma and rectitis tissue and that it may be an independent prognostic factor for rectal cancer.

## Introduction

Rectal cancer is one of the most common malignant tumors of the digestive system. The introduction of adjuvant chemoradiotherapy for the treatment of rectal cancer improved survival rates, but a high mortality rate is still associated with the malignancy. The main factors that affect the prognosis of patients with rectal cancer include tumor stage, the distance between the tumor and anus, and the circumferential resection margin [1–2]. Because similar patients can receive the same treatment yet ultimately have different outcomes, we hypothesize that there are other contributing factors.

Y-box binding protein-1 (YB-1) is the most evolutionarily conserved nucleic acid-binding protein known [3]. It is involved in the regulation of transcription and translation, DNA repair, alternative splicing of mRNA, RNA repair, cell proliferation, apoptosis, and drug resistance [4–7]. An increasing number of studies have demonstrated an association between YB-1 and various cancers. For example, this protein could drive the G1-S transition in tumor cells through the CDC6 pathway to promote proliferation [8]. YB-1 may act as a negative regulator of the tumor suppressor gene p53 and repressor of the p53 promoter [9]. YB-1 can form complexes with activating protein 2 and p53. Subsequent binding to the RE-1 response element of the matrix metalloproteinase 2 (MMP2) gene enhancer element activates the transcription of *MMP2*, thereby promoting tumor metastasis [10]. PIK3CA, epidermal growth factor receptor (EGFR), and mesenchymal-epithelial transition factor are modulated by YB-1 to promote the growth and survival of breast cancer cells [11–13]. YB-1 has also been associated with the resistance of tumor cells to therapeutics [14–15].

Several studies illustrated that YB-1 overexpression is associated with a poor prognosis for patients with malignant tumors such as head and neck cancer [16], non-Hodgkin lymphoma [17], breast cancer [18], osteosarcoma [19], non-small cell lung cancer [20], gastric cancer [21], and esophageal squamous cell carcinoma [22]. Currently, there are few reports on whether YB-1 expression affects the prognosis of patients with rectal cancer. Previous studies have illustrated that inflammation plays an extremely important role in the initiation and progression of cancer, including colorectal cancer[23]. The aims of this study were to evaluate the expression of YB-1 in non-neoplastic rectal tissue and rectal cancer tissue, and to evaluate the relationship between YB-1 expression and the prognosis of patients with rectal cancer.

## Materials and Methods

### Tissue samples and clinical data

The study was approved by the Medical Ethics Committee of Mianyang Central Hospital. All tissues were obtained with the consent of the patients, and written informed consent from the donors or the next of kin was obtained for use of the tissue samples for research purposes. For this study, 140 formalin-fixed, paraffin-embedded tissue specimens were obtained between 2008 and 2009 from the Department of Pathology at Mianyang Central Hospital. The specimens were collected from 140 patients, including 80 with rectal cancer, 30 with rectal tubular adenoma, and 30 with rectitis. All patients with rectal cancer underwent surgery, and after surgery, 69 of these patients received adjuvant chemoradiotherapy (radiation dose DT: 50 Gy/25 fractions; 5-fluorouracil [5-FU]–based chemotherapy regimen). The mean follow-up time was 46.8 months (range: 7–75 months). Eight patients with rectal cancer were lost to follow-up, and thus, clinical data were collected for 72 patients.

### Immunohistochemistry

Immunohistochemical analysis was performed as described previously [24]. Four-micrometer-thick formalin-fixed, paraffin-embedded tissue sections were deparaffinized, rehydrated in a gradient of alcohol, and subjected to a heat-induced epitope retrieval buffer consisting of Tris-EDTA at pH 7.4. The slides were then boiled for 3 min in a pressure cooker and then cooled in buffer to retrieve the epitopes. Slides were soaked in 3% H_2_O_2_ for 8 min and then washed with water. They were then incubated in a 1:200 dilution of a polyclonal rabbit antibody raised against human YB-1 (Cell Signaling Technology, Inc., Boston, MA, USA) for 1 h at room temperature. Following incubation with goat anti-rabbit secondary antibody, biotin horseradish peroxidase enzyme-labeled polymer from the Dako ChemMate EnVision Detection Kit (Dako Diagnostics Co. Ltd., Shanghai, China) was added. The chromogen was 3,3ʹ-diaminobenzidine (Dako Diagnostics Co. Ltd.). Finally, the sections were counterstained with hematoxylin. Breast cancer tissue slides were used as a positive control for YB-1 staining. A slide that was not treated with the primary antibody was used as a negative control. YB-1 immunopositivity was analyzed semiquantitatively. This method, developed by McCarty et al., is named the YB-1 histological score (HSCORE) [25]. The HSCORE accounts for both the intensity of staining and the percentage of cells that are stained for each level of staining intensity. The HSCOREs were obtained using the following algorithm: HSCORE = Ʃ (I × PC), where I and PC represent the intensity of staining and the percentage of cells that were stained for each level of staining intensity, respectively. Intensities were classified from zero (no staining) to three (very strong staining). All slides were examined and scored independently by three pathologists who were blinded to both the clinical and pathologic data. YB-1 immunopositivity was defined as the presence of any specific staining in the cytoplasm and/or nucleus.

### Statistical analysis

We performed statistical analysis using SPSS statistical software (IBM Corp., Armonk, NY, USA). The differential expression of YB-1 in tissue samples from patients with rectal rectitis, rectal tubular adenoma, and rectal cancer was analyzed using ANOVA statistical tests. ANOVA or *t*-tests were used to analyze the correlation between the clinicopathological parameters and YB-1 expression. A Cox proportional hazards regression model was used in a multivariate analysis of the impact of prognostic factors on survival. P values <0.05 were considered statistically significant in all tests.

## Results

### YB-1 expression in rectal cancer and non-neoplastic rectal tissues

**Fig 1:**
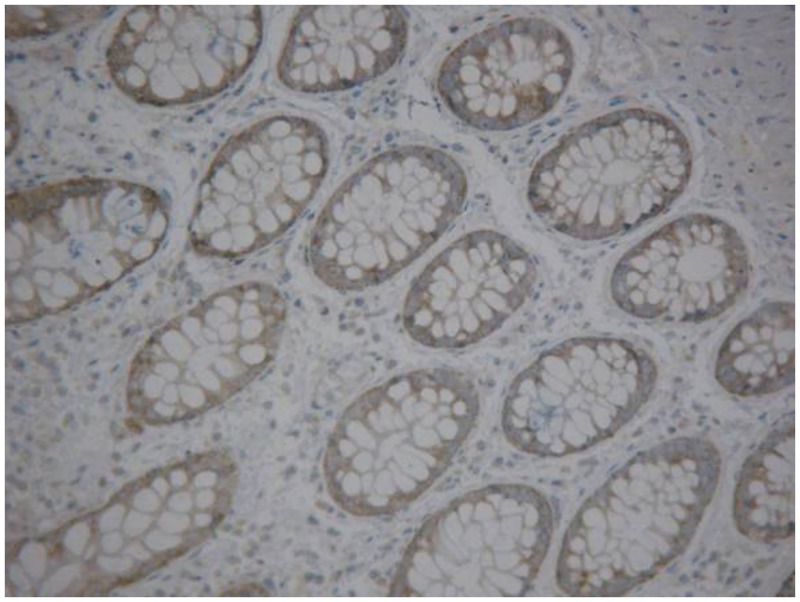
Immunohistochemical detection of Y-box binding protein-1 expression in rectitis tissue(200×).

**Fig 2:**
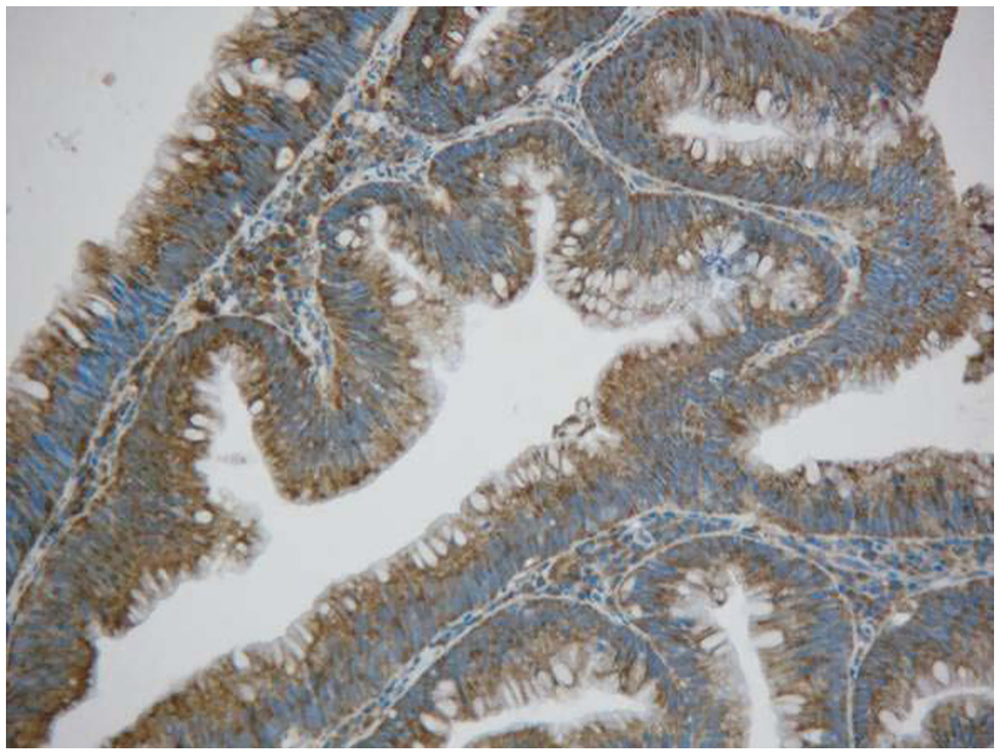
Immunohistochemical detection of Y-box binding protein-1 expression in rectal tubular adenoma tissue (200×).

**Fig 3:**
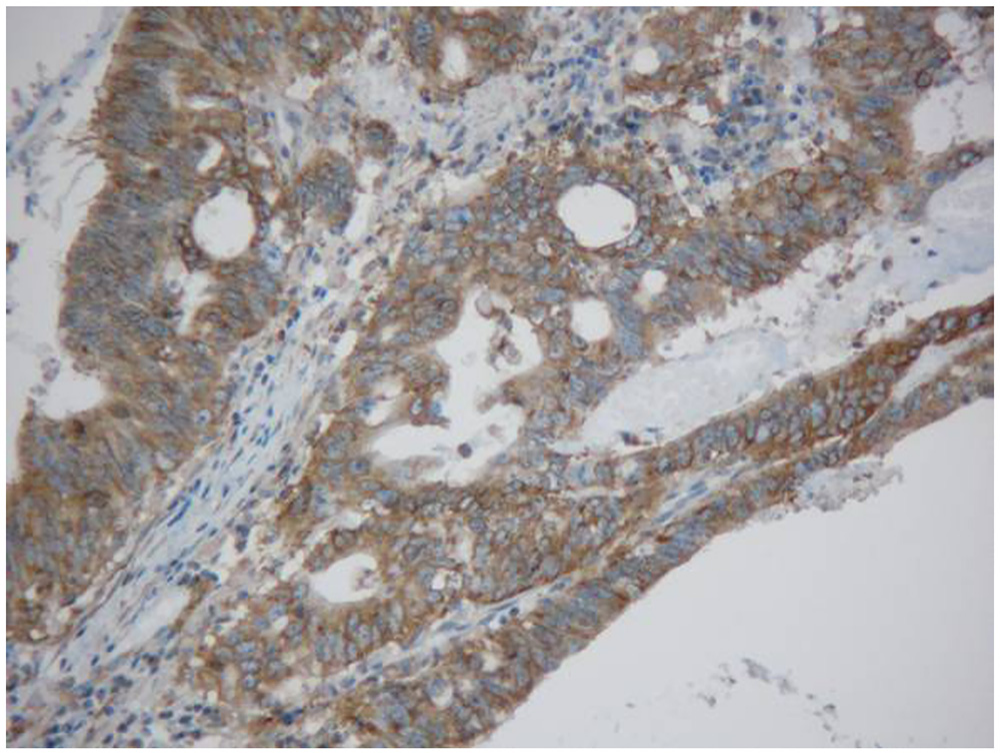
Immunohistochemical detection of Y-box binding protein-1 expression in rectal cancer tissue (200×).

Rectal cancer cells exhibited diffuse YB-1 staining in the cytoplasm (Figs. 1–3). In non-cancerous rectal tissue, only weak YB-1 staining was observed. Nuclear staining was rarely observed in any of the tissue samples. The mean YB-1 HSCOREs for rectal cancer, tubular adenoma, and rectitis tissue samples were 205.5, 164.3, and 137.7, respectively. YB-1 expression levels were higher in tissue from patients with rectal cancer than in non-neoplastic rectal tissue (P = 0.000; Table 1). However, YB-1 expression did not display any obvious correlation with patient age (P = 0.153), sex (P = 0.657), cancer stage (P = 0.245), or adjuvant chemoradiotherapy (P = 0.761). The clinical features of these patients are shown in Table 2.

**Table 1:** The differential expression of YB-1 in rectitis, rectal tubular adenoma and rectal cancer tissues.

Histologic diagnosis	HSCORE≥200(n)	HSCORE<200(n)	The mean of HSCORE	*P*
rectitis	0(0%)	30(100%)	137.7±52.3	0.000
rectal tubular adenoma	10(33.33%)	20(66.67%)	164.3±94.3	
rectal cancer	55(68.75%)	25(31.25%)	205.5±105.5	

**Table 2:** Clinicopathologic characteristics in 72 rectal cancer patients.

Clinicopathologic characteristics	n	YB-1 HSCORE		*P*
		≥200	<200	
The number of cases	72	50	22	
The average score of HSCORE	206.0	231.4	148.2	
Age				*P* = 0.153
≥60	37	23	14	
<60	35	27	8	
Sex				*P* = 0.657
Male	44	28	16	
Female	28	22	6	
Stage(AJCC2010)				*P* = 0.245
I	15	10	5	
II	47	31	16	
III	10	9	1	
Adjuvant chemoradiotherapy				*P* = 0.761
Yes	62	43	19	
No	10	7	3	
Differentiation				*P* = 0.660
Low	6	5	1	
Moderately	66	45	21	
High	0	0	0	
Lymph node metastasis				*P* = 0.026
Yes	10	10	0	
No	62	40	22	

### Survival analysis of YB-1 expression in rectal cancer

**Fig 4:**
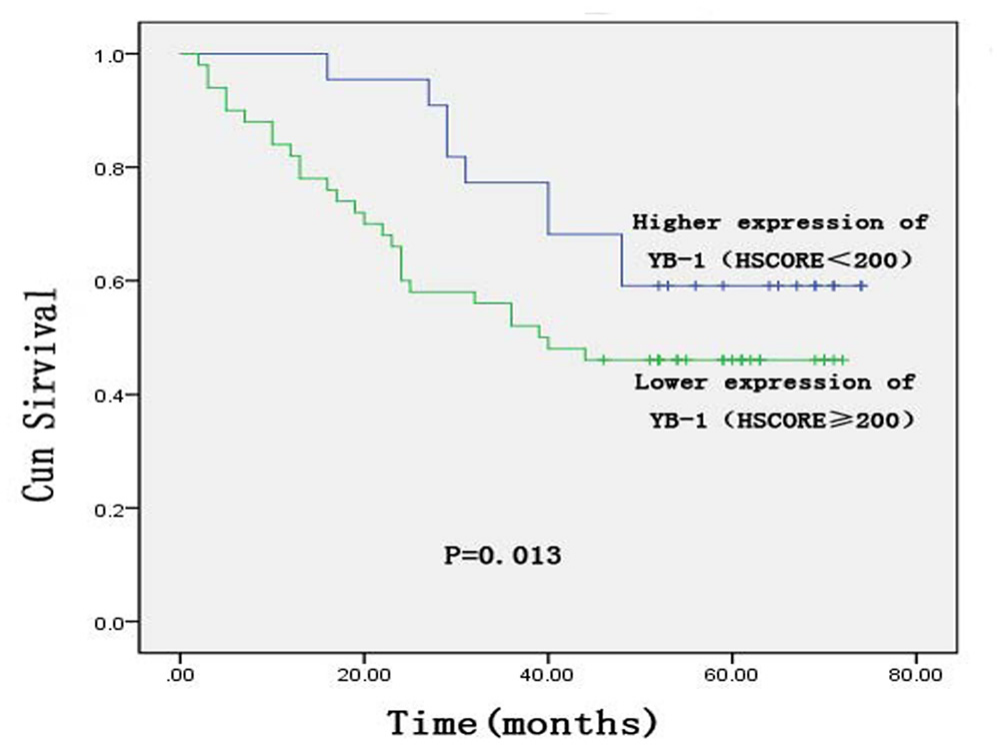
Kaplan—Meier curves of disease-free survival of the Y-box binding protein-1 expression status for patients with rectal cancer.

**Fig 5:**
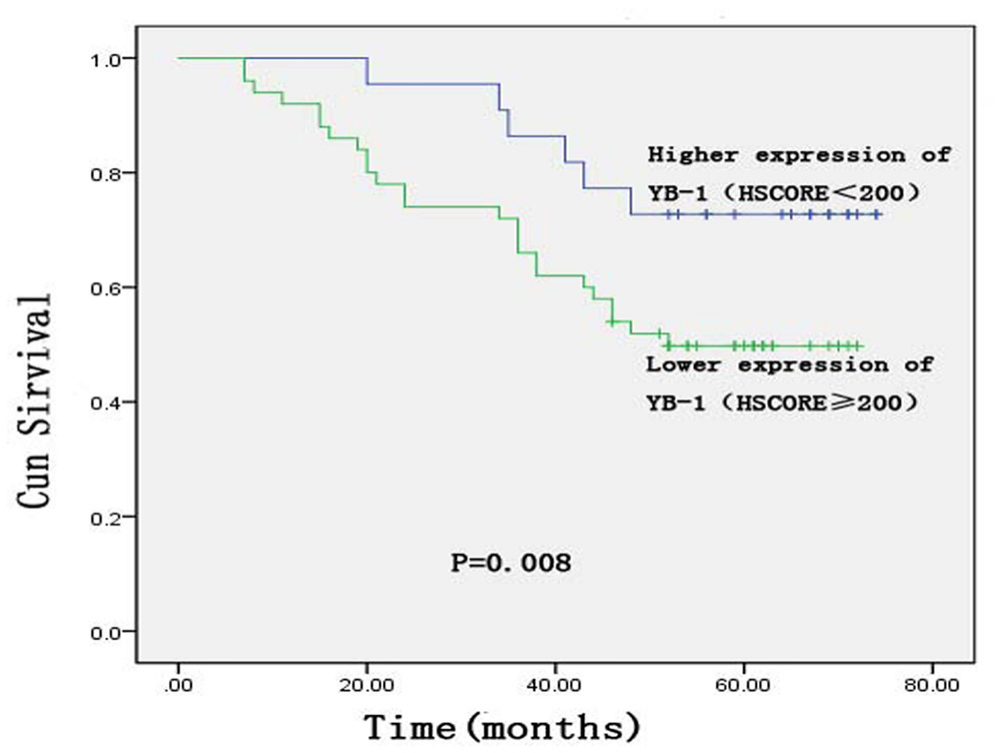
Kaplan—Meier curves of overall survival of the Y-box binding protein-1 expression status for patients with rectal cancer.

Higher YB-1 expression (HSCORE ≥ 200) was associated with poor disease-free survival (DFS) and overall survival (OS) (Figs. 4–5). The mean DFS was 38.2 months in 50/72 patients with rectal cancer, which was shorter than that of patients with lower YB-1 expression (HSCORE < 200). The mean DFS of patients with lower YB-1 expression was 52.4 months (P = 0.013). An analysis of OS revealed similar results. The mean OS time of patients with higher YB-1 expression (HSCORE ≥ 200) was 44.4 months, versus 57.3 months (P = 0.008) for patients with lower YB-1 expression (YB-1 HSCORE < 200).

The influence of clinical and pathologic characteristics on rectal cancer was analyzed in a multivariate analysis using a Cox’s proportional hazard model. The results indicated that clinical stage, adjuvant chemoradiotherapy, and YB-1 expression correlated with DFS and OS and that they may be independent prognostic factors that affect the survival times of patients with rectal cancer (Table 3).

**Table 3:** Multivariate analysis of clinicopathologic effectors on prognosis of cervical cancer patients.

Parameters	DFS			OS		
	*95%CI*	*HR*	*P*	*95%CI*	*HR*	*P*
Stage	2.774–171.978	21.9	0.003	2.733–186.442	22.574	0.004
Adjuvant chemoradiotherapy	0.114–0.892	0.319	0.029	0.07–0.69	0.207	0.004
YB-1	1.348–5.340	2.357	0.039	1.149–7.684	2.971	0.025

## Discussion

Many proteins are reported to be associated with the prognosis of patients with rectal cancer including vasohibin-1 [26] and miR-25 [27]. Few studies have focused on the association between YB-1 and rectal cancer using an immunohistochemical approach. Upregulation of YB-1 was observed in several tumors of the digestive system. For instance, YB-1 is overexpressed in the cytoplasm of esophageal squamous cell carcinoma cells [22]. Higher expression of YB-1 is also observed in the cytoplasm of gastric cancer cells, but nuclear staining is rarely observed [21]. In our study, we observed YB-1 expression in the cytoplasm of cells from three different tissues. The HSCORE for rectal cancer tissue was significantly higher than those for non-neoplastic rectal tissues, suggesting that *YB-1* may be a rectal cancer-associated oncogene.

YB-1 is an indicator of prognosis in several cancers. For example, the recurrence rate was higher in patients with nasopharyngeal carcinoma with high YB-1 expression than in patients with low expression of this protein [28]. In breast cancer, elevated nuclear YB-1 expression may be correlated with shorter DFS, disease-specific survival, and OS [29]. Our study revealed similar results. The mean DFS and OS of patients with higher expression of YB-1 were 38.3 and 44.4 months, respectively, versus 52.4 and 57.3 months, respectively, in patients with lower YB-1 expression. Patients with higher expression of YB-1 had shorter DFS (P = 0.013) and OS (P = 0.008) than those with lower YB-1 expression. Multivariate analysis of patient prognosis using a Cox proportional hazards model indicated that tumor stage, age, and sex had no correlation with prognosis but that YB-1 expression was an independent prognostic factor for rectal cancer.

The poor prognosis of patients with overexpression of YB-1 may be due to YB-1–induced resistance to chemoradiotherapy. In breast cancer cells, the activation of YB-1 at serine 102 mediates resistance to trastuzumab by increasing the number of CD44-positive cells [30]. YB-1 could modulate the reactivity of tumor cells to tamoxifen and fulvestrant by affecting the estrogen receptor (ER)-human EGFR2 signaling pathway in ER-positive breast cancer cells [31]. YB-1 can activate the multidrug resistance gene *MDR1* and the multidrug resistance-associated protein [32–35]. It can also induce resistance to mitomycin and cisplatin [36]. YB-1 is directly involved in the regulation of 5-FU—induced activation of the MVP promoter, which mediates drug resistance [37]. The chemotherapy regimen used in this study was based on platinum and 5-FU. We hypothesize that the poor patient prognosis could have resulted from drug resistance induced by YB-1 overexpression. Radiation-induced YB-1 phosphorylation promotes the repair of double-strand DNA breaks and post-irradiation survival through phosphatidylinositol 3-kinase/AKT and MAPK/extracellular signal-regulated kinase signaling pathways, which are downstream of EGFR signaling. In addition, depletion of YB-1 by siRNA could increase the sensitivity of breast cancer cells to radiation [38]. It is possible that similar mechanisms underlie the findings in our study. However, the mechanisms that underlie YB-1 overexpression in patients with rectal cancer and the functional consequences remain unclear.

## Conclusion

In conclusion, our study indicates that YB-1 expression is higher in rectal cancer tissue than in rectal tubular adenoma and rectitis tissue. Elevated expression of YB-1 may indicate a poor prognosis for patients with rectal cancer. A more detailed understanding of the signaling pathways in which YB-1 participates may result in the identification of new therapeutic targets for rectal cancer treatment.
